# 1355. The SARS-CoV-2 Monoclonal Antibody AZD3152 Potently Neutralizes Historical and Emerging Variants and is Being Developed for the Prevention and Treatment of COVID-19 in High-risk Individuals

**DOI:** 10.1093/ofid/ofad500.1192

**Published:** 2023-11-27

**Authors:** Joseph R Francica, Yingyun Cai, Seme Diallo, Kim Rosenthal, Kuishu Ren, Daniel J Flores, Andrew Dippel, Yuling Wu, Xiaoru Chen, Erin Cantu, Rakesh Choudhary, Michal Sulikowski, Hibret Adissu, Bhavna Chawla, Swagata Kar, Nydia van Dyk, Vaheh Oganesyan, Saravanan Rajan, Patricia C Ryan, Yueh-Ming Loo, Taylor Cohen, Mark T Esser, Wade Blair

**Affiliations:** AstraZeneca, Gaithersburg, Maryland; AstraZeneca, Gaithersburg, Maryland; AstraZeneca, Gaithersburg, Maryland; AstraZeneca, Gaithersburg, Maryland; AstraZeneca, Gaithersburg, Maryland; AstraZeneca, Gaithersburg, Maryland; AstraZeneca, Gaithersburg, Maryland; AstraZeneca, Gaithersburg, Maryland; AstraZeneca, Gaithersburg, Maryland; AstraZeneca, Gaithersburg, Maryland; AstraZeneca, Gaithersburg, Maryland; AstraZeneca, Gaithersburg, Maryland; AstraZeneca, Gaithersburg, Maryland; BIOQUAL Inc., Rockville, Maryland; BIOQUAL Inc., Rockville, Maryland; AstraZeneca, Gaithersburg, Maryland; AstraZeneca, Gaithersburg, Maryland; AstraZeneca, Gaithersburg, Maryland; AstraZeneca, Gaithersburg, Maryland; AstraZeneca, Gaithersburg, Maryland; AstraZeneca, Gaithersburg, Maryland; AstraZeneca, Gaithersburg, Maryland; AstraZeneca, Gaithersburg, Maryland

## Abstract

**Background:**

EVUSHELD was developed for the prevention and treatment of mild-to-moderate COVID-19 for high-risk individuals. However, the SARS-CoV-2 virus continues to evolve in the presence of natural- and vaccine-acquired immunity, escaping previously authorized antibody therapies such as bebtelovimab and EVUSHELD.

AZD3152 was selected to neutralize all known SARS-CoV-2 variants of concern and is being developed to provide immunocompromised individuals with continued protection against SARS-CoV-2.

**Methods:**

Structural analyses were performed to map the AZD3152 binding site. Neutralization assays were employed to determine the potency of AZD3152 across a panel of historical and emerging SARS-CoV-2 variants. Hamsters were prophylactically administered 6.7-60 mg/kg AZD3152 or a control antibody, then challenged intranasally with 6x10^3^ PFU WA-1 SARS-CoV-2, then monitored for weight loss; lung viral load and pathology were evaluated at days 3 and 7.

A 3 week GLP repeat IM and IV dose toxicity study in nonhuman primates (NHP) was performed.

**Results:**

AZD3152 binds to a conserved epitope on the spike receptor binding domain and blocks ACE2 binding. AZD3152 potently neutralizes authentic viral variants (EC_50_ range, 8.3 to 110.9 ng/mL) and SARS-CoV-2 pseudoviruses (EC_50_ range of 3.2 to 25.0 ng/mL), including XBB.1.5. Prophylactic administration of AZD3152 conferred dose-dependent protection to hamsters following challenge. Even at a suboptimal dose of 6.7 mg/kg, < 5% weight loss and 2 logs reduction in lung viral subgenomic RNA were observed. AZD3152 was not associated with any adverse findings in NHP and the no observed-adverse-effect-level was the highest dose tested (150 mg/kg). Toxicokinetics demonstrated similar systemic exposure following IM and IV dosing with bioavailability of 94.9% and half-life range of 15.2-26.5 days by IM.

**Conclusion:**

These results demonstrate that AZD3152 binds a conserved epitope resulting in broad neutralization of SARS-CoV-2 variants, protects hamsters from disease, and has a favorable safety profile in NHP. Thus, AZD3152 may serve as a next-generation antibody for the prevention and treatment of COVID-19.

**Disclosures:**

**Joseph R Francica, PhD**, AstraZeneca: Employee|AstraZeneca: Stocks/Bonds **Yingyun Cai, PhD**, AstraZeneca: Employee|AstraZeneca: Stocks/Bonds **Seme Diallo, MS**, AstraZeneca: Employee|AstraZeneca: Stocks/Bonds **Kim Rosenthal, MS**, AstraZeneca: Employee|AstraZeneca: Stocks/Bonds **Kuishu Ren, BS**, AstraZeneca: Employee|AstraZeneca: Stocks/Bonds **Daniel J. Flores, MS**, AstraZeneca: Employee|AstraZeneca: Stocks/Bonds **Andrew Dippel, PhD**, AstraZeneca: Employee|AstraZeneca: Stocks/Bonds **Yuling Wu, PhD**, AstraZeneca: Employee|AstraZeneca: Stocks/Bonds **Xiaoru Chen, PhD**, AstraZeneca: Employee|AstraZeneca: Stocks/Bonds **Erin Cantu, BS**, AstraZeneca: Employee|AstraZeneca: Stocks/Bonds **Rakesh Choudhary, MS**, AstraZeneca: Employee|AstraZeneca: Stocks/Bonds **Michal Sulikowski, PhD**, AstraZeneca: Employee|AstraZeneca: Stocks/Bonds **Hibret Adissu, PhD**, AstraZeneca: Employee|AstraZeneca: Stocks/Bonds **Nydia van Dyk, MSc**, AstraZeneca: Employee|AstraZeneca: Stocks/Bonds **Vaheh Oganesyan, PhD**, AstraZeneca: Employee|AstraZeneca: Stocks/Bonds **Saravanan Rajan, PhD**, AstraZeneca: Employee|AstraZeneca: Stocks/Bonds **Patricia C. Ryan, PhD**, AstraZeneca: Employee|AstraZeneca: Stocks/Bonds **Yueh-Ming Loo, PhD**, AstraZeneca: Employee|AstraZeneca: Stocks/Bonds **Taylor Cohen, PhD**, AstraZeneca: Employement|AstraZeneca: Stocks/Bonds **Mark T. Esser, PhD**, AstraZeneca: Employee|AstraZeneca: Stocks/Bonds **Wade Blair, PhD**, AstraZeneca: Employee|AstraZeneca: Stocks/Bonds

